# Level of Fear and Its Determinants in the Indian Population Due to COVID-19 Disease

**DOI:** 10.7759/cureus.28612

**Published:** 2022-08-31

**Authors:** Siddharth M Lodha, Shubho Acharya, Gurmeet Singh, Sumit Kumar, Sharanya Kohli, Pragya Sharma

**Affiliations:** 1 Department of Community Medicine, Maulana Azad Medical College, New Delhi, IND

**Keywords:** covid-19, healthcare worker, fear and anxiety, public mental health, fear of covid-19

## Abstract

Background

The novel coronavirus disease 2019 (COVID-19) infection was declared a global health emergency by the World Health Organization. A total of three waves across most of the states in India have been reported to date, during which strict lockdown was imposed and conditional relaxations were offered between the subsequent waves. Amid the high morbidity and mortality, there has been severe psychological distress among people which has led to mental health impairment.

Methodology

We investigated the level of fear in the Indian population due to COVID-19 using the Fear of COVID-19 Scale (FCS-19) and various factors influencing it. A cross-sectional study was undertaken across India among participants more than 18 years of age by recruiting participants through social media platforms such as WhatsApp and Instagram. Along with the FCS-19 questionnaire, sociodemographic information about the participants, preexisting history of comorbidities, and psychiatric illnesses were collected. The study sample was drawn by convenience technique, and the data were collected over two months from October 2021 to December 2021.

Results

A total of 419 participants (212 females and 207 males) participated in the study. The mean FCS-19 score of the population was 18.29 ± 6.43 (SD). Participants with a history of COVID-19-related deaths in their own family or surrounding areas had a significantly higher FCS-19 score than those without a history of COVID-19-related deaths. The mean FCS-19 score for healthcare professionals was also significantly lower than for other professions. FCS-19 scores were significantly higher among participants with psychiatric conditions than those without.

Conclusions

The study showed a positive association between a preexisting mental health disease and FCS-19 score and a negative association if the participant was a healthcare professional. While other factors such as age, gender, residential area, and preexisting comorbidity did not show a significant association with fear associated with COVID-19.

## Introduction

Since the novel coronavirus disease 2019 (COVID-19) infection was declared a global health emergency by the World Health Organization (WHO) on January 30, 2020, it has significantly impacted people’s physical and mental health [1]. The first confirmed case was reported in December 2019 from Wuhan, China, which later spread worldwide, with the first case detected in India in January 2020. Consequently, the first nationwide lockdown was initiated in March 2020, intended for 21 days but stretched significantly longer as COVID-19 cases continued to rise. A total of three waves have been reported to date, during which strict lockdown was imposed and conditional relaxations were offered between the subsequent waves. Amid the high morbidity and mortality, repetitive cycles of lockdown resulting in the loss of jobs and disruption of businesses have led to substantial financial losses, resulting in psychological distress and mental health impairment. Repetitive lockdowns have caused a surge in the number of victims of domestic violence as they cannot get away from their abusers [2].

Schools and colleges were shut down, leading to disruption in education, reduced physical activity besides loss of friends and classmates, resulting in increased boredom, lack of personal space at home, disruption of daily routines, increased parental pressure to study, and increased family violence, all of which contributed to increased anxiety, anger, depression, and other forms of mental distress [3].

Quarantine and self-isolation, the measures people had to undertake after they got infected by COVID-19, also affected their livelihood and increased feelings of loneliness and anxiety. Fear of getting infected and stress regarding family members’ health led to constant stress among common people. Due to the highly contagious nature of the virus, regular hand washing and masks were the important preventive actions mandated to break the chain of transmission. Repetitive thoughts of virus in the surroundings leading to repeated, compulsive hand washing and sanitizing cycles also led to a higher predisposition to obsessive-compulsive disorder (OCD) [4].

Because COVID-19 had higher mortality in older adults, concern about their own health, besides their family members’ health and the disease’s financial impact, contributed to psychological stress in older adults. Moreover, this age group experienced loneliness, age discrimination, and excessive worry. Studies have indicated that the female and elderly populations have been under severe psychological stress, which was way higher than expected [5].

The increasing number of cases also posed an increase in demand for the health system and a lot of pressure and burnout in healthcare professionals. Healthcare professionals have increased depression and anxiety, severely impacting their quality of life (QoL) [6].

Overall, panic and stress have led to poor mental health conditions across all age groups and professions. Thus, this study was conducted to assess the level of fear among people due to COVID-19 and the different factors and patterns that contributed to anxiety and mental health issues.

## Materials and methods

Study design and participants

A cross-sectional study was undertaken across most states in India among participants over 18 years of age and both genders from October 2021 to December 2021 using social media. As the research was conducted during the COVID-19 pandemic, social media was preferred to ensure proper social distancing. The sample was drawn by convenience sampling, and the data were collected over two months from October 2021 to December 2021.

Data collection and procedure

Data were collected on a semi-structured questionnaire, pretested and authenticated along with a validated Fear of COVID-19 Scale (FCS-19) [7] questionnaire through social media platforms such as WhatsApp, Gmail, and Instagram. The questionnaire was divided into two parts. The first part covered the consent and sociodemographic details of the participants, along with some other specific information regarding medical history, COVID-19 vaccination, etc. The second part included the FCS-19. A total of 419 people responded to the questionnaire.

The FCV-19S, based on the Protection Motivation Theory, is an open-source questionnaire with a unidimensional factor structure. The FCV-19S has been confirmed to have reliability and validity in various countries such as Bangladesh, Iran, Israel, Italy, New Zealand, Russia and Belarus, Saudi Arabia, Turkey, and Vietnam. It has come to have more widespread use than other coronavirus-related measures. Results obtained using FCV-19S can be associated with various factors, including sociodemographic and residential environments.

The participants indicate their level of agreement with the statements using a five-item Likert-type scale. Answers included strongly disagree, disagree, neutral, agree, and strongly agree. The minimum score for each question is 1, and the maximum is 5. A total score can be calculated by adding each item score (ranging from 7 to 35).

Statistical analysis

The data were analyzed using the SPSS for Windows version 26.0 (IBM Corp., Armonk, NY, USA). The descriptive data analysis used numbers, percentages, arithmetic mean, standard deviation (SD), and minimum and maximum values. The relationship between the independent groups with normal distribution was analyzed using the independent sample t-test and association tests. The significance level for statistical analysis was accepted as a P-value of <0.05.

Ethical consideration

Ethical permission was taken from the Institutional Ethics Committee of Maulana Azad Medical College to conduct the study (F.1/IEC/MAMC/87/05/2021/No.523). The participants were informed about the motive of the study and data confidentiality, and only after taking their consent, data were collected.

## Results

Of the 419 study participants, 50.6% were females. The majority (50.1%) of the respondents were aged between 20 and 29 years, followed by more than 50 years (15%), between 40 and 49 years (13.4%), 30-39 years (13.4%), and ≤20 years (13.4%) (N = 41). More than half (52.3%) of the participants were students, followed by service, including both government and private jobs (30.8%), business (9.1%), and 1.2% were unemployed. The majority (91.6%) of the respondents were urban dwellers. Of these, 44.2% were from Delhi, followed by Haryana (20.5%), Uttar Pradesh (6.9%), Karnataka, and West Bengal (4.8%). Less than one-fifth (18.6%) of the study participants were from other states. The majority (79%) of the respondents were not healthcare professionals (Table 1).

**Table 1:** Sociodemographic characteristics of the study participants. *Homemakers, retired. **Assam, Bihar, Gujarat, Himachal Pradesh, Jammu and Kashmir, Jharkhand, Kerala, Lakshadweep, Madhya Pradesh, Maharashtra, Odisha, Punjab, Rajasthan, Tamil Nadu, Telangana, Uttrakhand. ***Doctors, nurses.

Characteristics		N = 419	Percentage (%)
Age groups	≤19 years	41	9.8
	20–29 years	210	50.1
	30–39 years	49	11.7
	40–49 years	56	13.4
	≥50 years	63	15.0
Sex	Male	207	49.4
	Female	212	50.6
Occupation	Student	219	52.3
	Service (government + private)	129	30.8
	Business	38	9.1
	Unemployed	5	1.2
	Others*	28	6.6
Place of residence	Urban	384	91.6
	Rural	35	8.4
State of residence	Delhi	185	44.2
	Haryana	86	20.5
	Uttar Pradesh	29	6.9
	Karnataka	21	5.0
	West Bengal	20	4.8
	Other states**	78	18.6
Healthcare professional***	Yes	88	21
	No	331	79

The vaccination preference of the participants varied. Overall, 44.9% of the participants thought all of them were equally effective, 33.7% went with Covishield, 13.4% with Covaxin, and 6.4% with Sputnik. The majority (49.9%) of the participants relied on social media for information regarding COVID-19, 20.8% on newspapers, and 29.4% on television (Table 2).

**Table 2:** Vaccination preference and the source of information regarding COVID-19 in the study population. COVID-19: coronavirus disease 2019

Category	Subcategory	N = 419	Percentage (%)
Preferred COVID-19 vaccine	Covaxin	56	13.4
	Covishield	141	33.7
	Sputnik V	27	6.4
	All are effective	188	44.9
	None of them are effective	7	1.7
The major source of information regarding COVID-19	Newspaper	87	20.8
	Social media	209	49.9
	Television	123	29.4

Out of all the participants, 9.8% suffered from hypertension, 7.2% from diabetes, 5.5% from hypothyroidism, and 4.5% from obesity (Table 3).

**Table 3:** Preexisting comorbidities in the study population. *Steroid use, asthma, patients on chemotherapy, polycystic ovarian disorder, heart problem. **Attention-deficit hyperactivity disorder, bipolar disorder, complex post-traumatic stress disorder.

Comorbidities	N = 419	Percentage (%)
Diabetes	30	7.2
Hypertension	41	9.8
Hypothyroidism	23	5.5
Obesity	19	4.5
Others*	16	3.7
Anxiety	26	6.2
Depression	32	7.6
Other mental health conditions**	9	2.14

The mean FCS-19 score of the population was 18.29 ± 6.43 (SD). Participants with a history of COVID-19-related deaths in their family or surrounding areas had a significantly higher FCS-19 score than those without a history of COVID-19-related deaths. The mean FCS-19 score for healthcare professionals was also significantly lower than for other professions. FCS-19 scores were significantly higher among participants with psychiatric conditions than those without (Table 4).

**Table 4:** T-test analysis of various factors and their association with the mean FCS-19 score. *Doctors, nurses. FCS-19: Fear of COVID-19 Scale; COVID-19: coronavirus disease 2019

Factors	Sub-factors	Mean FCS-19 score	P-value
Sex	Male	18.63	0.291
	Female	17.96	
Area	Rural	19.20	0.384
	Urban	18.20	
Death due to COVID-19 in the family or area	Yes	18.82	0.037
	No	17.48	
Healthcare professional*	Yes	16.42	0.002
	No	18.79	
Health insurance	Yes	18.46	0.342
	No	17.76	
Comorbidity	Yes	17.52	0.195
	No	18.51	
Psychiatric condition	Yes	21.28	0.000
	No	17.82	
Employment status during the pandemic	Yes	18.30	0.981
	No	18.28	
Smoking	Yes	18.91	0.386
	No	18.17	

## Discussion

This study aimed to determine the level of fear of COVID-19 and its determinants in the Indian population by employing the FCS-19 scale. Specifically, the study investigated the association of the level of fear with various factors such as age, gender, presence of a comorbid condition, residential area, occupational status, smoking, and history of preexisting mental illnesses such as depression, anxiety, and bipolar disorder. Further, it was also analyzed whether any death in the family or neighborhood or occupation influenced the level of fear. Several studies have shown that the female gender is more predisposed to experience anxiety and various other forms of mental disorders [8-10]. In previous studies using the FCS-19 scale, some studies showed a positive association with the female gender [11] while others showed no association [12,13]. However, in our study, there was no significant association between the FCS-19 score and gender. This can be due to the timeframe in which the study was conducted. During this time, the cases had decreased, there was no state of emergency/lockdown, and the fear had subsided with the evolution of the pandemic across the last three waves. Research has shown that college students are generally more vulnerable to the impact of COVID-19 [14]. This study did not show a significant deviation in the score of students compared to other population segments, probably because of more urban participation, which included a more aware population regarding COVID-19 implications and vaccination. Several studies have shown a significant exacerbation of fear and anxiety among healthcare workers [15-19]. Our study has shown results contradicting the previous studies. The mean FCS-19 score of the healthcare workers was significantly lower compared to the remaining population. It might be due to the continuous exposure of the healthcare workers for the past two years to the COVID-19 pandemic and its ramifications that have led to desensitization to the fear and anxiety associated with their close work with infected patients. In addition, our research was conducted during a low infectivity period, and by this time, healthcare workers had adapted to the panic and fear and were duty-bound toward patient care. The primary source of information of the participant did not significantly impact the fear, which may be due to the lack of any significant difference between the information passed on by different platforms regarding COVID-19. However, a study showed that people who relied on news on television as a significant source of information had a greater level of fear and anxiety [12]. Smoking did not have any significant effect on the fear of COVID-19. However, one research that used the FCS-19 scale showed a positive association with smoking [20]. This can be due to the study design and tool, which limits participation from rural areas. The level of fear among participants with preexisting comorbid conditions was almost similar to those with no comorbid conditions, which shows that comorbidity does not increase the levels of fear in the study participants. However, some studies have shown that an underlying comorbid condition significantly impacts the outcome associated with the infection [21,22]. However, another study conducted at the beginning of the pandemic contradicts our findings, showing a positive association between underlying comorbidity and fear and anxiety [23]. History of pre-existing mental health illnesses (such as depression, anxiety, and bipolar disorder) is positively associated with FCS- 19 scores. This is because the person is already in a more vulnerable state and in times of severe distress with high mortality occurring globally due to COVID-19 might have created a sense of insecurity and fear in an individual who is already at high risk. Various other studies have also shown that people with previous psychiatric illness history showed significant exacerbation with the onset of the pandemic, which was associated with aggravated fear [24-27].

This study was limited by the questionnaire being only available in English; thus, participation was limited to the English-comprehending section of the population. Along with this, there was a disproportionate level of participation from people residing in urban areas, which might have caused the study to show a decreased level of fear when compared to the ground level, as the urban population segment tends to be more aware and has access to better healthcare facilities compared to the rural segment. To avoid physical contact during the time of the pandemic, the participants were only recruited through online modes, which might have impacted the sample size of the study.

## Conclusions

This study showed that having a preexisting mental health illness significantly impacted fear and can create an increased level of anxiety and a feeling of vulnerability in a person. Even though this study was conducted after the culmination of the second wave of COVID-19 in India, when cases had significantly dipped, the fear had not decreased in proportion to the cases and situation in participants with a preexisting mental health illness. Thus, a more thorough evaluation is needed in this population segment to recognize the impact of the pandemic on this vulnerable section, and more awareness among the general population not affected by any mental health illness to help the affected people around them in times of such uncertainty. The negative association of fear when the participant works in the healthcare sector shows that people in this sector have calmed down after the initial exponential increase in workload and unpredictability due to the lack of information about the novel COVID-19. This decrease in fear will probably lead to better patient management and handling of the situation. The decrease in fear shows the healthcare industry’s increased hold on the pandemic with declining cases and mortality across the globe.
